# Supplementary material for: The effectiveness of global protected areas for climate change mitigation
Source: Nat Commun. 2023 Jun 1;14:2908. doi: 10.1038/s41467-023-38073-9 (PMC10235066; doi:10.1038/s41467-023-38073-9)
Supplement: Supplementary file 1 — Supplementary Information [file 41467_2023_38073_MOESM1_ESM.pdf]

Supplementary Materials for

Title: The Effectiveness of Global Protected Areas for Climate Change Mitigation

Authors: L. Duncanson[1]*, M. Liang[1], V. Leitold[1], J. Armston[1], S. M. Krishna Moorthy[1], R. Dubayah[1], S. Costedoat[2], B. J. Enquist[3], L. Fatoyinbo[4], S. J. Goetz[5], M. Gonzalez-Roglich[6], C. Merow[7], P. R. Roehrdanz[2], K. Tabor[4,8], A. Zvoleff[2]

Affiliations:

[1]Department of Geographical Sciences, University of Maryland, College Park

[2] Moore Center for Science, Conservation International, Arlington, VA 22202, USA

[3]Department of Ecology and Evolutionary Biology, University of Arizona, Tucson, AZ 85721, USA. and The Santa Fe Institute, 1399 Hyde Park Rd., Santa Fe, NM 87501, USA.

[4]NASA Goddard Space Flight Center

[5] School of Informatics, Computing and Cyber Systems, Northern Arizona University

[6] WCS Argentina

[7] Eversource Energy Center and Department of Ecology and Evolutionary Biology, University of Connecticut, Storrs, CT, USA

[8] Department of Geography and Environmental Systems, University of Maryland Baltimore County, Baltimore, MD, USA

*Corresponding author. Email: lduncans@umd.edu

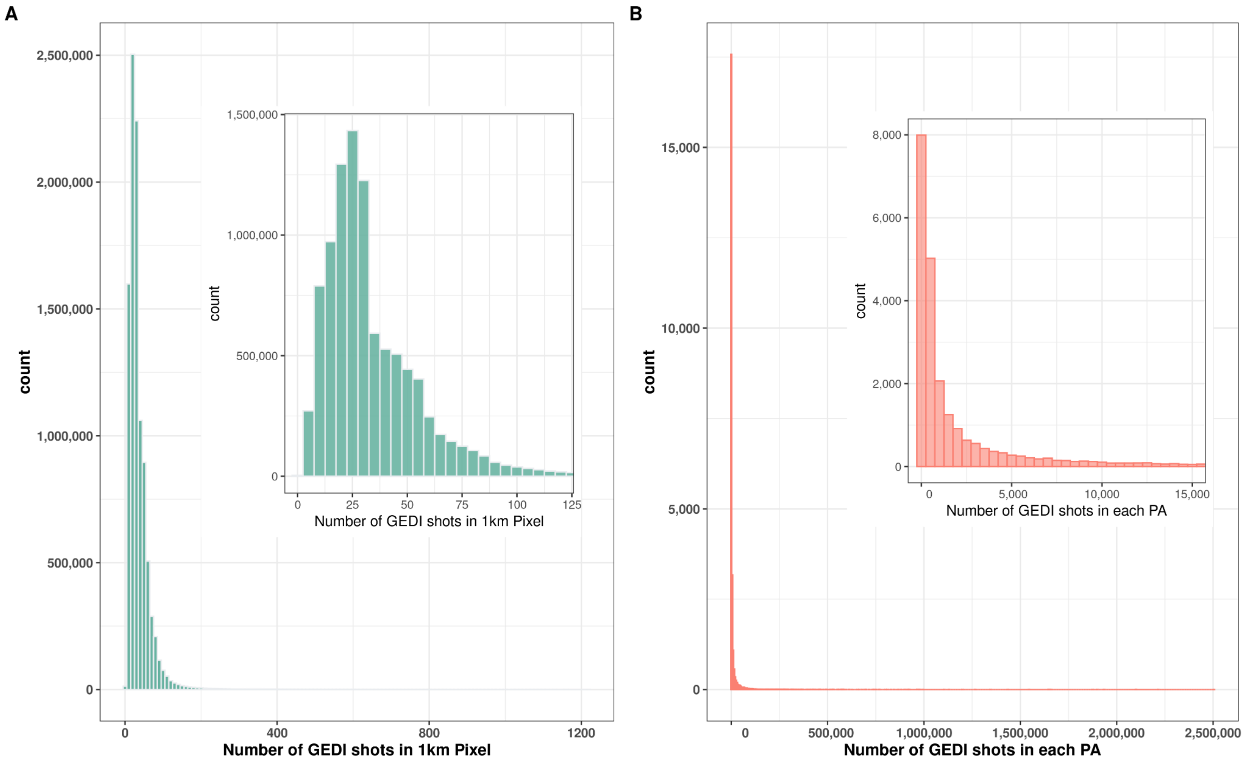

Supplementary Fig. 1. The distribution of GEDI samples within 1 km pixels analyzed in this study. GEDI enables a richer analysis of forest structure than has previously been possible, as demonstrated by the distribution of GEDI samples in each matched 1 km pixel (A) and PA (B). The largest PA in this analysis had >2 million GEDI samples (Shot in cell has median of 28, and shot in PA median is 639).

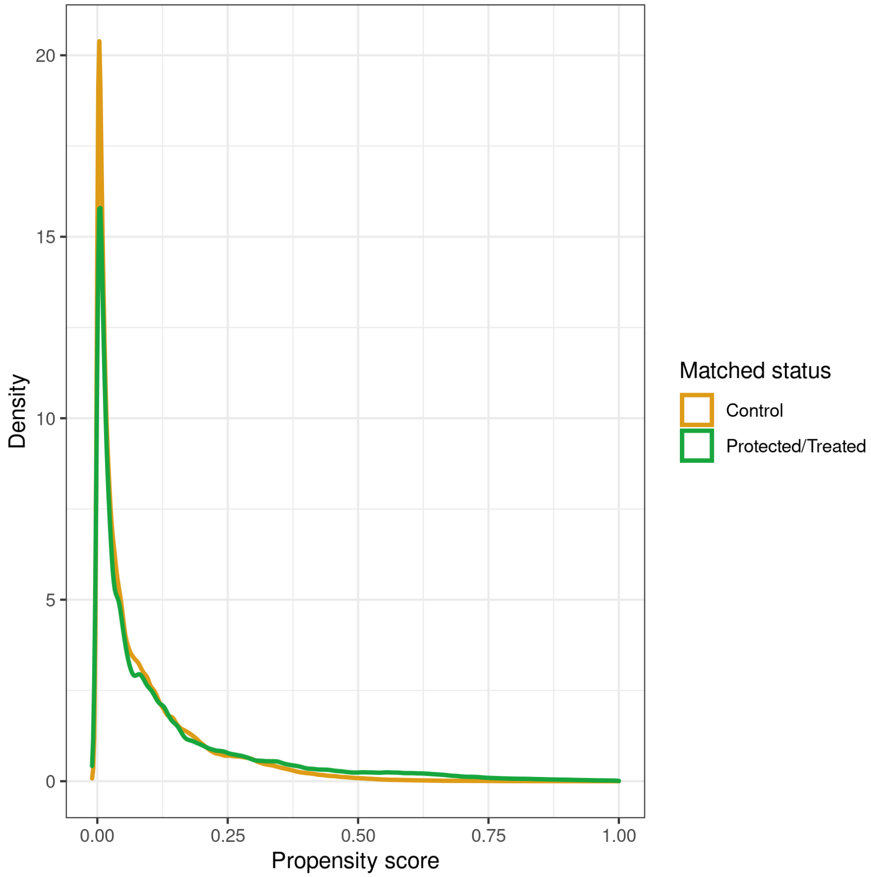

Supplementary Fig. 2. Matching algorithm propensity scores. The distribution of propensity scores of the matched protected area pixels and unprotected matched pixels for all matched PAs, with PA pixels in green and counterfactual pixels in orange.

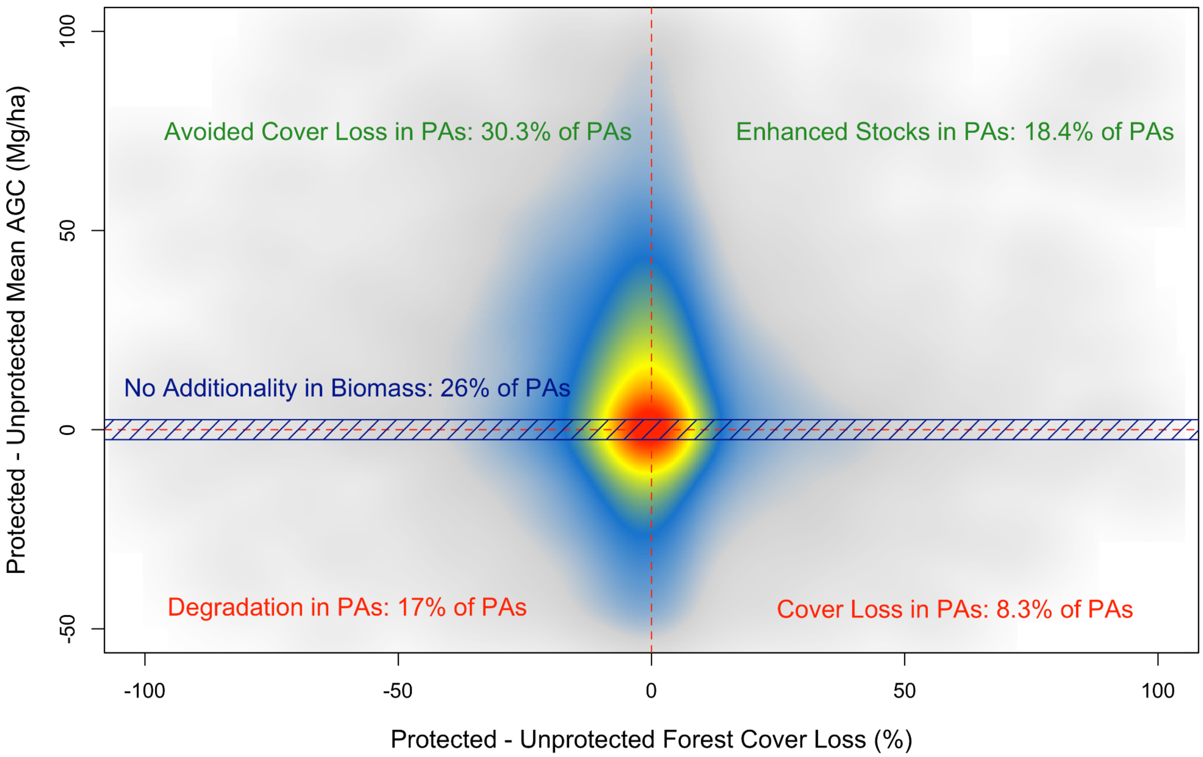

Supplementary Fig. 3. Domain-wide comparison of differences between PA and counterfactual AGB and forest cover loss. More than half of the analyzed PAs had more than 5 Mg/ha average higher AGBD than unprotected counterfactuals, around a quarter exhibited no difference in AGBD, and about a quarter had increased deforestation or degradation within the PAs. We consider less than 2.5 Mg/ha difference to be negligible or no additionality in biomass.

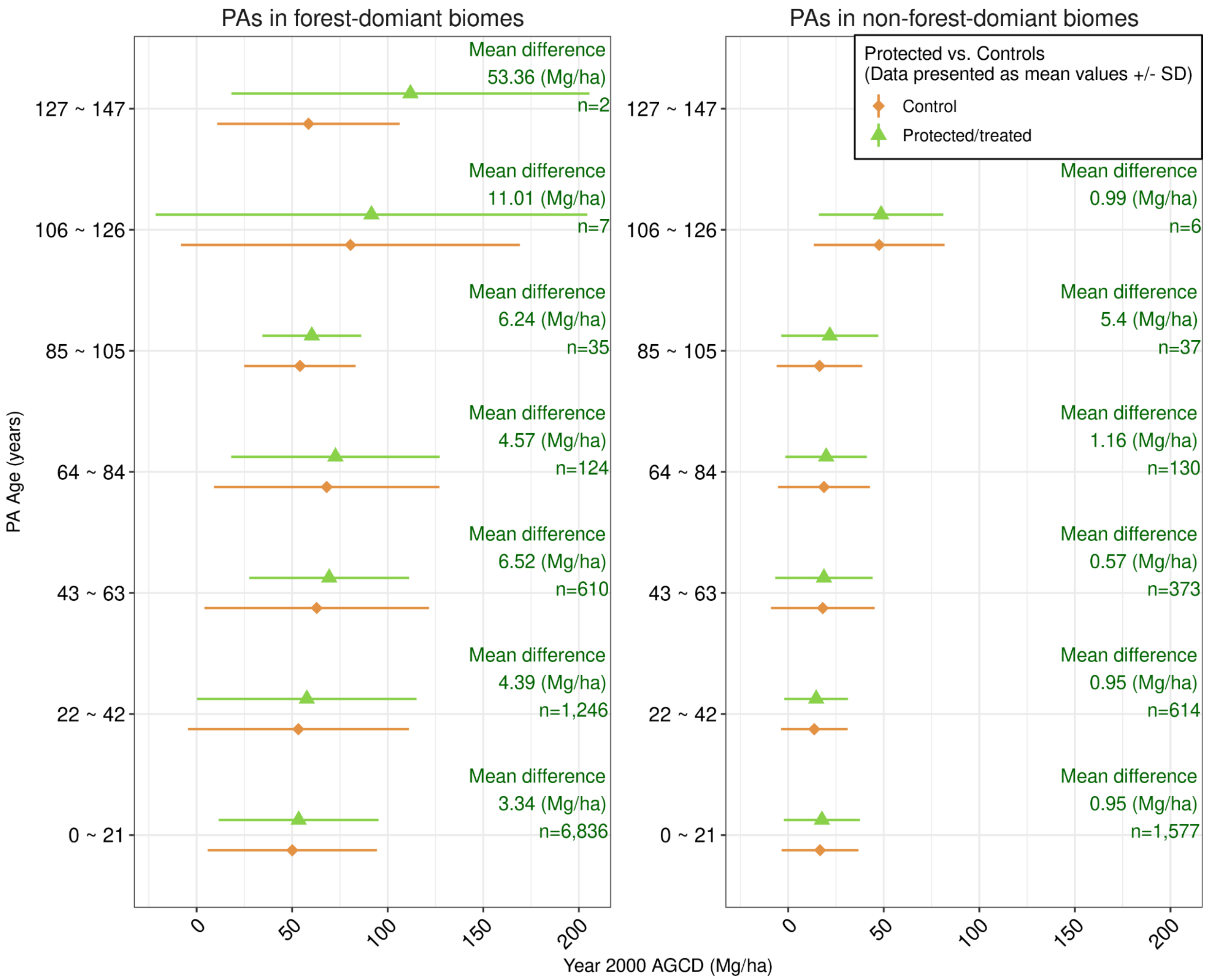

Supplementary. Fig. 4. Relationship between PA ages and the mean difference in PA and matched unprotected sites with respect to the year 2000 AGCD. Recently established PAs are represented as PA ages (0~21) and these PAs have the lowest mean difference between matched PA sites and control sites, indicating lower capacity to preserve AGCD.

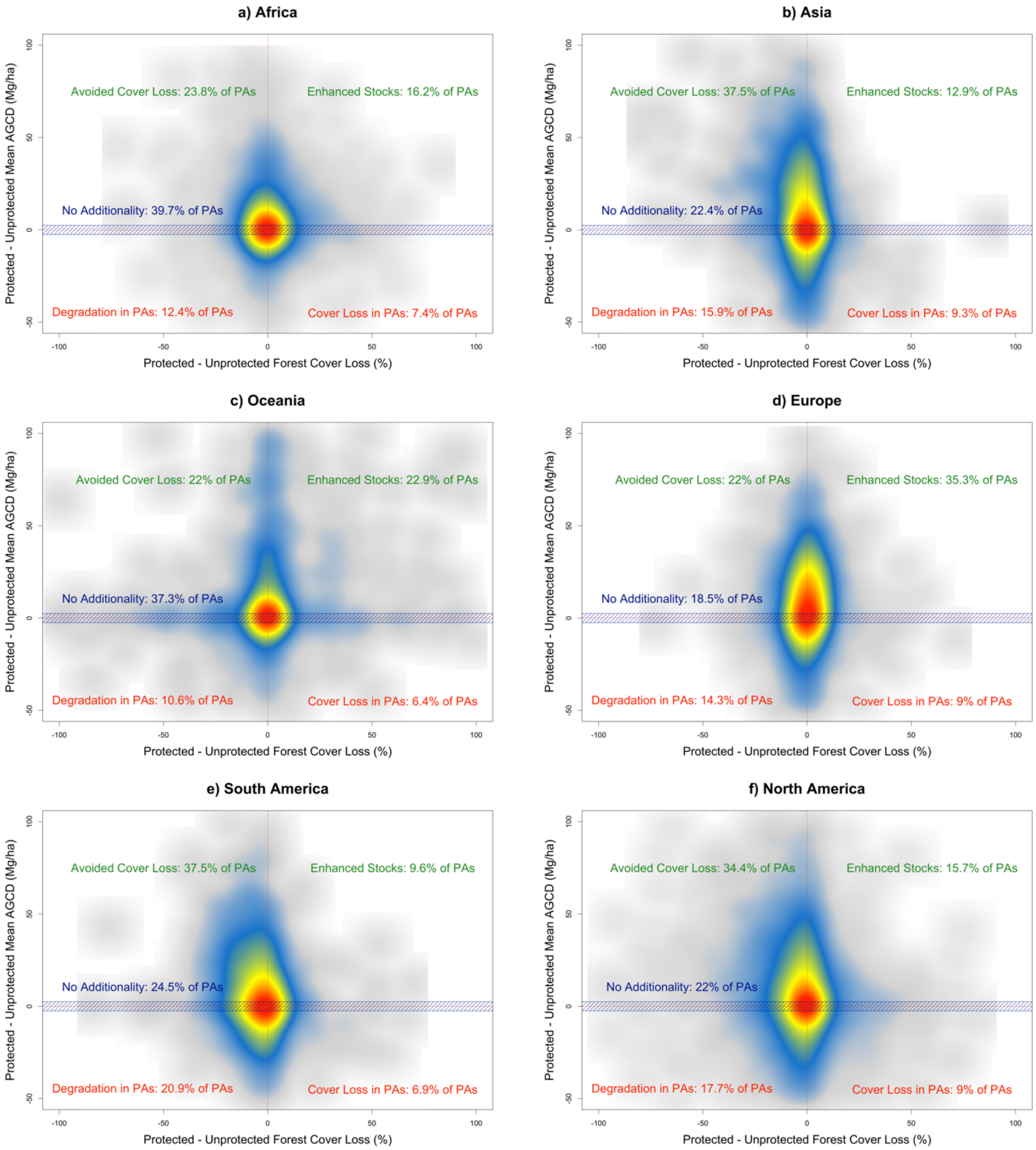

Supplementary Fig. 5. Continental comparison of differences between PA and counterfactual AGB and forest cover loss. The attribution of AGC results to either carbon effectiveness (through avoided deforestation, degradation and regrowth) varied by continent. Notably, South America and Asia had the highest rates of avoided emissions from forest loss, Africa and Oceania had the largest proportions of PAs with no additionality in AGC, and rates of degradation and forest loss within PAs were relatively consistent across continents. These density plots are colored with respect to data density, ranging from grey (least data) to red (most data). We consider less than 2.5 Mg/ha difference to be negligible or no additionality in biomass.

Supplementary Table 1. Datasets used in this study, and covariates used for matching in year 2000 baseline conditions.

Usage	Data	Time	Details and sources
Primary data for analysis	World Database on Protected Areas (WDPA)	09/2020	UNEP-WCMC and IUCN, www.protectedplanet.net
	GEDI	04/2019-10/2020	Level 2A, Level 2B, and Level 4A https://lpdaac.usgs.gov/
Exact matching	Land cover	2000	ESA CCI aggregated to UNCCD 7 land cover classes [1]
	Ecoregion	2001	Terrestrial Ecoregions of the World (TEOW), WWF [2]
	Biome		
Propensity score matching	Population density	2000	Gridded Population of World Version 4 (GPWv4) [3]
	Population count		
	Mean temperature	1990-1999	WorldClim Vl Bioclimatic variables [4]
	Mean precipitation		
	Elevation	2000	
	Slope		CGIAR-CSI SRTM
	Distance to cities	--	Conservation International [5]
	Travel time to cities	2000	Global Accessibility Map, EU Science Hub [6]

Supplementary References

1. ESA. Land Cover CCI Product User Guide Version 2. Tech. Rep. (2017). Available at: maps.elie.ucl.ac.be/CCI/viewer/download/ESACCI-LC-Ph2-PUGv2_2.0.pdf
2. Olson, D. M. *et al.* Terrestrial Ecoregions of the World: A New Map of Life on Earth: A new global map of terrestrial ecoregions provides an innovative tool for conserving biodiversity. *Bioscience* **51**, 933–938 (2001).
3. Center for International Earth Science Information Network - CIESIN - Columbia University. 2018. Gridded Population of the World, Version 4.11 (GPWv4): Population Count, Revision 11. Palisades, NY: NASA Socioeconomic Data and Applications Center (SEDAC). https://doi.org/10.7927/H4JW8BX5. Accessed Apr. 10th, 2020.

4. Hijmans, R. J., Cameron, S. E., Parra, J. L., Jones, P. G. & Jarvis, A. Very high resolution interpolated climate surfaces for global land areas. *Int. J. Climatol.* **25**, 1965–1978 (2005).
5. Hewson, J., Crema, S. C., González-Roglich, M., Tabor, K. & Harvey, C. A. New 1 km Resolution Datasets of Global and Regional Risks of Tree Cover Loss. *Land* **8**, 14 (2019).
6. Nelson, A. Estimated travel time to the nearest city of 50,000 or more people in year 2000. *Ispra, Italy* (2008).
