# Supplementary material for: The effectiveness of global protected areas for climate change mitigation
Source: Nat Commun. 2023 Jun 1;14:2908. doi: 10.1038/s41467-023-38073-9 (PMC10235066; doi:10.1038/s41467-023-38073-9)
Supplement: Supplementary file 3 — Reporting Summary [file 41467_2023_38073_MOESM3_ESM.pdf]

nature portfolio

Corresponding author(s): Laura Duncanson

Last updated by author(s): Apr 5, 2023

Reporting Summary

Nature Portfolio wishes to improve the reproducibility of the work that we publish. This form provides structure for consistency and transparency in reporting. For further information on Nature Portfolio policies, see our Editorial Policies and the Editorial Policy Checklist.

Statistics

For all statistical analyses, confirm that the following items are present in the figure legend, table legend, main text, or Methods section.

n/a	Confirmed	
☐	☒	The exact sample size (*n*) for each experimental group/condition, given as a discrete number and unit of measurement
☒	☐	A statement on whether measurements were taken from distinct samples or whether the same sample was measured repeatedly
☐	☒	The statistical test(s) used AND whether they are one- or two-sided<br>*Only common tests should be described solely by name; describe more complex techniques in the Methods section.*
☐	☒	A description of all covariates tested
☐	☒	A description of any assumptions or corrections, such as tests of normality and adjustment for multiple comparisons
☐	☒	A full description of the statistical parameters including central tendency (e.g. means) or other basic estimates (e.g. regression coefficient) AND variation (e.g. standard deviation) or associated estimates of uncertainty (e.g. confidence intervals)
☐	☒	For null hypothesis testing, the test statistic (e.g. *F*, *t*, *r*) with confidence intervals, effect sizes, degrees of freedom and *P* value noted<br>*Give P values as exact values whenever suitable.*
☒	☐	For Bayesian analysis, information on the choice of priors and Markov chain Monte Carlo settings
☒	☐	For hierarchical and complex designs, identification of the appropriate level for tests and full reporting of outcomes
☐	☒	Estimates of effect sizes (e.g. Cohen's *d*, Pearson's *r*), indicating how they were calculated

Our web collection on statistics for biologists contains articles on many of the points above.

Software and code

Policy information about availability of computer code

Data collection	No software was used in data collection.
Data analysis	Code for analyzing the data presented in the paper (algorithm development, figure generation, etc.) are publicly available at git repo: https://github.com/lauraduncanson/GEDI_PA.git and the version used in this paper is archived at https://zenodo.org/badge/latestdoi/284058541.

For manuscripts utilizing custom algorithms or software that are central to the research but not yet described in published literature, software must be made available to editors and reviewers. We strongly encourage code deposition in a community repository (e.g. GitHub). See the Nature Portfolio guidelines for submitting code & software for further information.

Data

Policy information about availability of data

All manuscripts must include a data availability statement. This statement should provide the following information, where applicable:

- Accession codes, unique identifiers, or web links for publicly available datasets
- A description of any restrictions on data availability
- For clinical datasets or third party data, please ensure that the statement adheres to our policy

All data used in this study are from publicly available sources. GEDI data are archived on NASA Distributed Active Archive Centers (DAACs). GEDI's footprint-level height data were taken from the GEDI02_A height and elevation product, available at LPDAAC: 10.5067/GEDI/GEDI02_A.002. GEDI's PAI and cover data were taken from the GEDI02_B product also available at LPDAAC: 10.5067/GEDI/GEDI02_B.002. Finally, GEDI's footprint-level biomass (AGBD) data were taken from the GEDI04_A. The WDPA database can be downloaded at www.protectedplanet.net. For the matching variables used in the preprocessing, the 2000 land cover

products can be downloaded at http://maps.elie.ucl.ac.be/CCI/viewer/download.php.The WWF ecoregions and biomes can be downloaded at https://www.worldwildlife.org/publications/terrestrial-ecoregions-of-the-world. The gridded population datasets are retrieved from https://doi.org/10.7927/H4JW8BX5. The annual mean precipitation and temperature datasets are processed from the WorldClim version 1 datasets downloaded from https://developers.google.com/earth-engine/datasets/catalog/WORLDCLIM_V1_MONTHLY#description. Elevation and slope are processed using CGIAR SRTM downloaded from https://developers.google.com/earth-engine/datasets/catalog/CGIAR_SRTM90_V4. Distance to cities dataset is retrieved from https://doi.org/10.3390/land8010014. Travel time to cities dataset can be downloaded from https://forobs.jrc.ec.europa.eu/products/gam/download.php. For additional details related to the matching variables, see supplementary Table 1. Intermediate datasets such as preprocessed matching results are available upon request.

Human research participants

Policy information about studies involving human research participants and Sex and Gender in Research.

Reporting on sex and gender	Not applicable.
Population characteristics	Not applicable.
Recruitment	Not applicable.
Ethics oversight	Not applicable.

Note that full information on the approval of the study protocol must also be provided in the manuscript.

Field-specific reporting

Please select the one below that is the best fit for your research. If you are not sure, read the appropriate sections before making your selection.

☐ Life sciences ☐ Behavioural & social sciences ☒ Ecological, evolutionary & environmental sciences

For a reference copy of the document with all sections, see nature.com/documents/nr-reporting-summary-flat.pdf

Ecological, evolutionary & environmental sciences study design

All studies must disclose on these points even when the disclosure is negative.

Study description	We analyzed ~412,000,000 lidar waveform samples from NASA's GEDI mission at a global scale. Samples were aggregated to a 1 km resolution, and 1 km cells were matched within global Protected Areas (PAs) and ecologically similar unprotected 1 km cells. GEDI samples within and outside of PAs were then compared to determine differences in forest structure between the two sets.
Research sample	We did not sample from the full population - we used all quality-filtered data to conduct this global analysis. The only reduction in sample size from the GEDI dataset related to data quality (i.e. the application of mission quality filter flags to remove erroneous data, e.g. associated with clouds or laser signal contamination). The mission collects data as samples instead of continuous mapping due to engineering constraints on satellite lidar observation, but collects data at a global scale.
Sampling strategy	No sampling was conducted, all quality-filtered GEDI data were used.
Data collection	NASA's GEDI emits lidar pulses from the International Space Station, and each pulse illuminates ~25 m diameters on the Earth's surface, and bounces back to the ISS for recording. The time elapsed between emission and return of the beam provides surface elevation, and the within-beam reflection provides 3D canopy structure. NASA Goddard Space Flight Center (GSFC) managed the collection of all GEDI data from the ISS.
Timing and spatial scale	Data for this study were from the first 18 months of on-orbit GEDI collection, between April 2019 and October 2020. Data were global-scale under the ISS coverage (+/- 52 degrees latitude).
Data exclusions	No data were excluded except for lidar quality-filtering (e.g. from cloud contamination).
Reproducibility	All analysis was automated and run on a high end computing processing cluster. The results were re-generated by multiple team members to ensure consistency in the findings.
Randomization	This is not applicable to this study, as there was no sampling involved for the analysis of GEDI data
Blinding	This is not applicable to this study, as there was no sampling involved for the analysis of GEDI data

Did the study involve field work? ☐ Yes ☒ No

Reporting for specific materials, systems and methods

We require information from authors about some types of materials, experimental systems and methods used in many studies. Here, indicate whether each material, system or method listed is relevant to your study. If you are not sure if a list item applies to your research, read the appropriate section before selecting a response.

Materials & experimental systems

n/a	Involved in the study
x	Antibodies
x	Eukaryotic cell lines
x	Palaeontology and archaeology
x	Animals and other organisms
x	Clinical data
x	Dual use research of concern

Methods

n/a	Involved in the study
x	ChIP-seq
x	Flow cytometry
x	MRI-based neuroimaging
